# Supplementary material for: Factors associated with community acquired severe pneumonia among under five children in Dhaka, Bangladesh: A case control analysis
Source: PLoS One. 2022 Mar 23;17(3):e0265871. doi: 10.1371/journal.pone.0265871 (PMC8942236; doi:10.1371/journal.pone.0265871)
Supplement: Attachment — Submitted filename: Response to Reviewers.doc [file pone.0265871.s001.doc]

**Reviewer #1:**

**Comment:**

Abstract: 'annual incidence' in line 22 and 'each year' on line 23 are a repetition.

**Response:** Thank you very much for spotting this. We have deleted “each year” from line 24 as suggested.

**Comment:**

Introduction
- since you are reporting absolute incidences, it would be useful to also indicate the global change in the population of the children referred to in line 49 (i.e. the denominator for those incidences).

**Response:** Thank you so much for the suggestion. We have edited line 49, it now reads “Implementation of feasible and effective interventions has reduced under-five pneumonia death substantially from 13·6 per 1000 livebirths in 2000 to 6·6 per 1000 livebirths in 2015” (line 61).

**Comment:**

- would it be accurate to use 'non-severe pneumonia' to refer to what you currently call 'pneumonia'; for example, in line 70: "we analysed a large dataset of children with severe and non-severe pneumonia..." and in line 71/72: "... factors associated with severe pneumonia compared to non-severe pneumonia."

**Response:** We appreciate and accept your suggestion. We have replaced all “non-severe pneumonia” term to “pneumonia” (line 112, 258).

**Comment:**
Methods
- please report the methods according to STROBE guidelines for case-control studies (see [https://www.equator-network.org/wp-content/uploads/2015/10/STROBE_checklist_v4_case-control.pdf](https://linkprotect.cudasvc.com/url?a=https%3A%2F%2Fwww.equator-network.org%2Fwp-content%2Fuploads%2F2015%2F10%2FSTROBE_checklist_v4_case-control.pdf&c=E,1,jYQXomntjkuS0I0hBWqF8zIdIG89d2X9lUXfQb2zLUTEGPGuD3BCJHHRgcmj636qUUfTdhAOnNKzRDCQ6bP-3s1Z02kyQoXAV7pGbK47-u6qAlFRBQ,,&typo=1&ancr_add=1)).

**Comment:** Thank you so much for the excellent suggestion. Please find the STROBE table below. We have mentioned the line numbers where the information was specified in the manuscript.

|  | Item No | Recommendation | Checked |
| --- | --- | --- | --- |
| **Title and abstract** | 1 | (*a*) Indicate the study’s design with a commonly used term in the title or the abstract | Line 1 and 19 |
|  |  | (*b*) Provide in the abstract an informative and balanced summary of what was done and what was found |  |
| Introduction | | |  |
| Background/rationale | 2 | Explain the scientific background and rationale for the investigation being reported | Lines 50-80 |
| Objectives | 3 | State specific objectives, including any prespecified hypotheses | Lines 82-86 |
| Methods | | |  |
| Study design | 4 | Present key elements of study design early in the paper | Lines 105-112 |
| Setting | 5 | Describe the setting, locations, and relevant dates, including periods of recruitment, exposure, follow-up, and data collection | Lines 95-101 |
| Participants | 6 | (*a*) Give the eligibility criteria, and the sources and methods of case ascertainment and control selection. Give the rationale for the choice of cases and controls | Lines 134-157 |
|  |  | (*b*) For matched studies, give matching criteria and the number of controls per case |  |
| Variables | 7 | Clearly define all outcomes, exposures, predictors, potential confounders, and effect modifiers. Give diagnostic criteria, if applicable | Lines 195-223 |
| Data sources/ measurement | 8* | For each variable of interest, give sources of data and details of methods of assessment (measurement). Describe comparability of assessment methods if there is more than one group | Lines 105-112 |
| Bias | 9 | Describe any efforts to address potential sources of bias |  |
| Study size | 10 | Explain how the study size was arrived at | Lines 111-112 |
| Quantitative variables | 11 | Explain how quantitative variables were handled in the analyses. If applicable, describe which groupings were chosen and why |  |
| Statistical methods | 12 | (*a*) Describe all statistical methods, including those used to control for confounding | Lines 181-193 |
|  |  | (*b*) Describe any methods used to examine subgroups and interactions | Line 190-193 |
|  |  | (*c*) Explain how missing data were addressed |  |
|  |  | (*d*) If applicable, explain how matching of cases and controls was addressed |  |
|  |  | (*e*) Describe any sensitivity analyses |  |
| Results | | |  |
| Participants | 13* | (a) Report numbers of individuals at each stage of study—eg numbers potentially eligible, examined for eligibility, confirmed eligible, included in the study, completing follow-up, and analysed | Line 230  Fig 1. |
|  |  | (b) Give reasons for non-participation at each stage |  |
|  |  | (c) Consider use of a flow diagram |  |
| Descriptive data | 14* | (a) Give characteristics of study participants (eg demographic, clinical, social) and information on exposures and potential confounders | Lines 245-250  Table 1. |
|  |  | (b) Indicate number of participants with missing data for each variable of interest |  |
| Outcome data | 15* | Report numbers in each exposure category, or summary measures of exposure | Lines 258-264 |
| Main results | 16 | (*a*) Give unadjusted estimates and, if applicable, confounder-adjusted estimates and their precision (eg, 95% confidence interval). Make clear which confounders were adjusted for and why they were included |  |
|  |  | (*b*) Report category boundaries when continuous variables were categorized |  |
|  |  | (*c*) If relevant, consider translating estimates of relative risk into absolute risk for a meaningful time period |  |

**Comment:**

- there doesn't seem to be any value on the chi-squared tests, Mann-Whitney tests or t-tests listed in line 175 given your intention to conduct univariable regression models as described in line 177.
**Response:** Thank you for your comment. To get the p-values in table 1, We used the chi-squared tests, Mann-Whitney tests, or t-tests. However, as per your other suggestion, we are keeping Table 1 pure descriptive, so, we have deleted the line from data analysis section.

**Comment:**

Results
- linked to the last comment in methods (above), the results described in line 246 relating to unadjusted regression analysis would be expected to be identical to those of the univariate test p-values in Table 1. I would recommend that you keep table 1 purely descriptive with no hypothesis tests of the differences between the two groups and p-values, as this is what table 2 explores.

**Response:** Authors agree with the suggestion. We have edited Table 1 and kept it purely descriptive as per your valuable suggestion. Find the table below (Line 245).

**Table 1. Comparison of characteristics of the study population with severe pneumonia (cases) and pneumonia (controls) according to the WHO classification^1^**

| **Variables** | **Severe Pneumonia**  **n=1693 (%)** | **Pneumonia**  **n=904(%)** |
| --- | --- | --- |
| **Presenting clinical features** |  |  |
| Duration of illness > 3days | 1536 (67.2) | 751 (32.8) |
| Temperature ≥ 38 °C | 701 (41.4) | 176 (19.5) |
| Pulse rate/min, mean (SD) ^b^ | 140.9 (16.8) | 139.0 (15.8) |
| **Nutritional status** |  |  |
| MUAC^a^, mean (SD) | 13.7 (1.2) | 14.0 (1.2) |
| Severe underweight (<-3SD) | 125 (7.4) | 55 (6.2) |
| Severe stunting (<-3SD) | 221 (13.1) | 97 (10.9) |
| **Sociodemographic profile** |  |  |
| Gender (male) | 1066 (63.0) | 510 (56.4) |
| **Child’s age** |  |  |
| 2-11 mo. | 1040 (61.4) | 494 (54.7) |
| 12-23 mo. | 408 (24.1) | 255 (28.2) |
| 23-59 mo. | 245(14.5) | 155 (17.6) |
| **Exclusive breastfed** | 858 (50.7) | 490 (54.2) |
| **Immunization status** |  |  |
| Pentavalent | 1599 (94.5) | 855 (94.6) |
| PCV | 1466 (86.6) | 759 (84.0) |
| Measles | 211/439 (48.1) | 336/644 (52.8) |
| **Father’s education** |  |  |
| Illiterate | 252 (15.0) | 157 (18.1) |
| Primary | 483 (28.7) | 254 (29.3) |
| Secondary | 677 (40.2) | 311 (35.9) |
| Higher | 274 (16.3) | 144 (16.6) |
| **Mother’s/caregiver’s education** |  |  |
| Illiterate | 189 (11.2) | 131 (14.6) |
| Primary | 522 (30.9) | 321 (35.9) |
| Secondary | 773 (45.7) | 338 (37.8) |
| Higher | 207 (12.2) | 105 (11.7) |
| **Household member** |  |  |
| ≥5 | 829 (49.0) | 436 (48.3) |
| **Number of siblings** |  |  |
| Two or more | 1020 (60.3) | 549 (60.8) |
| **Socio-economic context** |  |  |
| **Household income in BDT, Median (IQR)** ^c^ | 19000 (14000, 30000) | 15000 (12000, 24000) |
| **Wealth index** |  |  |
| Wealthiest | 383 (22.6) | 125 (13.9) |
| Wealthier | 374 (22.1) | 154 (17.1) |
| Middle | 326 (19.3) | 181 (20.1) |
| Poorer | 312 (18.4) | 215 (23.9) |
| Poorest | 297 (17.6) | 225 (25.0) |
| **Received care** |  |  |
| Private | 492 (39.2) | 97 (25.1) |
| Public | 156 (12.4) | 21 (5.4) |
| Pharmacy | 555 (44.3) | 244 (63.2) |
| Others | 51 (4.1) | 24 (6.2) |
| **Type of delivery** |  |  |
| Caesarean section | 752 (44.4) | 356 (39.4) |
| **Environment and household hygiene** |  |  |
| Treatment of Drinking water, safe | 1036 (61.2) | 467 (51.7) |
| Toilet Facility, improved**^d^** | 1019 (60.2) | 411 (45.5) |
| **Predominant wall in the house** |  |  |
| Brick | 1347 (79.6) | 639 (70.7) |
| **Floor materials** |  |  |
| Cement | 1430 (84.5) | 734 (81.1) |
| **Cooking fuel source** |  |  |
| Wood | 182 (10.8) | 139 (15.4) |
| Gas | 1557 (92.0) | 777 (86.1) |
| **Co-morbidities** |  |  |
| Diarrhoea | 65 (3.8) | 24 (2.7) |

^a^MUAC= Mid Upper Arm Circumference in cm; ^b^SD= Standard Deviation; ^c^ IQR= Inter Quartile Range;

**^d^** improved= flush+ pour flush+ ventilated pit latrine

^1^Revised WHO classification and treatment of childhood pneumonia at health facilities 2014

**Comment:**
- in table 2, you should report a single likelihood ratio p-value or global wald test for multicategorical variables such as wealth index and received care, not the individual wald test p-values comparing each category to the reference category as currently done. It is that single p-value that should be used to assess evidence of association with the whole variable. For example, you report that received care was no longer associated with the outcome after adjusting for other variables, but this is unlikely to be correct based on a single global test (as described above).

**Response:** We appreciate your suggestion. We re-analyzed the data according to your suggestion and presented a single likelihood ratio p-value for the categorical variables (wealth index, received care). Find the table below (Line 258).

**Table 2. Factors associated with severe pneumonia compared to pneumonia among under-five children**

| **Risk factors** | **Unadjusted OR (95% CI)** | **p-value** | ***Adjusted OR (95% CI)** | **p-value** |
| --- | --- | --- | --- | --- |
| **Presenting clinical features** |  |  |  |  |
| **Duration of illness** |  |  |  |  |
| <3 days | Reference |  | Reference |  |
| ≥3 days | 1.13 (1.09, 1.16) | <0.001 | 1.55 (1.19, 2.02) | 0.001 |
| **Temperature ≥ 38 °C** |  |  |  |  |
| No | Reference |  | Reference |  |
| Yes | 2.92 (2.41, 3.54) | <0.001 | 2.66 (2.17, 3.26) | <0.001 |
| **Severe stunting** |  |  |  |  |
| No | Reference |  | Reference |  |
| Yes | 1.23 (0.95, 1.58) | 0.109 | 1.44 (1.09, 1.91) | 0.009 |
| **Sociodemographic profiles** |  |  |  |  |
| **Child's sex** |  |  |  |  |
| Female | Reference |  | Reference |  |
| Male | 1.31 (1.11, 1.55) | 0.001 | 1.33 (1.11, 1.60) | 0.002 |
| **Wealth index** |  |  |  |  |
| Wealthiest | Reference | <0.001 | Reference | <0.001 |
| Wealthier | 0.79 (0.6, 1.04) |  | 0.58 (0.41, 0.83) |  |
| Middle | 0.58 (0.44, 0.77) |  | 0.49 (0.34, 0.70) |  |
| Poorer | 0.47 (0.36, 0.61) |  | 0.41 (0.28, 0.59) |  |
| Poorest | 0.43 (0.33, 0.56) |  | 0.47 (0.31, 0.70) |  |
| **Received care** |  |  |  |  |
| Private | Reference | <0.001 | Reference | <0.001 |
| Public | 1.46 (0.88, 2.42) |  | 1.28 (0.70, 2.33) |  |
| Pharmacy | 0.45 (0.34, 0.58) |  | 0.47 (0.34, 0.65) |  |
| Others | 0.18 (0.14, 0.23) |  | 0.22 (0.16, 0.29) |  |

Abbreviations: OR= Odds Ratio, CI= Confidence Interval

*Adjusted for temperature, duration of illness, pulse rate, child’s age, child’s sex, stunting, received prior medical care, wealth index, mother’s education, cooking fuel source- gas, wood

Results of unadjusted and adjusted odds ratio were calculated using simple and multivariable logistic regression

**Reviewer #2:**

**Comment:**

The presence of crepitation indicating alveolar involvement is part of the clinical diagnosis of pneumonia. It is possible that its absence in the control group may have occurred due to differential diagnoses of other respiratory diseases. I suggest removing the presence of crepitation as a risk factor.

**Response:** Thank you so much for your advice. We have re-analyzed the data by removing the crepitation variable as a risk factor from the multivariable model that you have suggested (Line 258).

**Comment:**

Regarding size, it can be as the authors did, although in our opinion it would be more appropriate to respect the case-control (1:1 ratio) study design and the cases and controls had the same number of participants

**Response:** Thank you so much for your suggestion. We did the secondary data analysis with all the available samples in the study. The study sample size was determined based on the original study objective. Children presenting to study clinics were assessed and those diagnosed as severe pneumonia, N=1963, and pneumonia, N=904 were included in this analysis. We kept these total estimated sample sizes and analyzed the data accordingly. Therefore, we have deleted the sample size section from the manuscript as it appeared to be unnecessary.
