# Supplementary material for: Factors associated with community acquired severe pneumonia among under five children in Dhaka, Bangladesh: A case control analysis
Source: PLoS One. 2022 Mar 23;17(3):e0265871. doi: 10.1371/journal.pone.0265871 (PMC8942236; doi:10.1371/journal.pone.0265871)
Supplement: Attachment — Submitted filename: Response to Reviewers.doc [file pone.0265871.s002.doc]

**Cover letter**

Date: 27 February 2022

To: Dr. Emily Chenette

Editor-in-Chief

PLOS ONE

From: Dr. A S G Faruque

Corresponding author,

Manuscript ID: PONE-D-21-06226R1

Subject: Response to the comments of the reviewers of PLOS ONE Journal Manuscript ID: PONE-D-21-06226R1 entitled “Factors associated with community-acquired severe pneumonia among under-five children in Dhaka, Bangladesh: A case-control analysis”.

Dear Ms. Chenette,

Thank you for providing us the opportunity to resubmit our manuscript following revision. We greatly appreciate helpful and precise comments from the academic editor and respected reviewer. We have attempted to address them in full. We now submit the revised manuscript, which highlights the changes in track change and also a clean version, as well as this cover letter which provides a point-by-point response to the reviewers’ comments. We would like to mention here that this manuscript has neither been published nor submitted for publication elsewhere and will not be submitted for publication to any other journal while this manuscript would be under consideration for publication by PLOS ONE. We sincerely hope that this manuscript is now suitable for publication in your esteemed journal.

Thank you

**Comments from the Academic Editor:**

**Comments:**

Please address the remaining comments from reviewer 1 regarding operational definitions.

Please also remove reference to crepitation in the conclusion of the abstract.

I think there does still need to be a section on sampling in your methods where you describe how the final sample was obtained and clarifying that you included all eligible children.

**Response:** Thank you so much for your kind review and valuable suggestions. As suggested, we have edited the ‘operational definitions’ as per reviewer 1’s suggestion in lines 126-133.

We have removed ‘crepitation’ in the conclusion of the abstract (line 50).

We appreciate your suggestion on adding a section on sampling in method section. We have added and now it reads, “Sample size:The secondary data analysis was done with the available samples in the study. Children presenting to study clinics were assessed and those diagnosed as severe pneumonia, N=1963, and pneumonia, N=904 were included in this analysis” in lines 116-119.

**Reviewer #1:**

**Comment:**

Reviewer #1: Please edit 'operational definitions' paragraph in lines 118 to 124 as follows for clarity: "Pneumonia was defined as a history of cough or difficult breathing and lower chest wall in-drawing or age-specific fast breathing (≥50 and ≥40 breaths/minute for 2–11 month-olds and 12–59 month-olds respectively) without any danger signs [13,14]. Severe pneumonia was defined as pneumonia with at least one of the following danger signs: central cyanosis or hypoxemia (oxygen saturation < 90% measured by pulse oximeter), severe respiratory distress (e.g. grunting, very severe chest in-drawing), inability to breastfeed or drink, lethargy or unconscious, or convulsion."

**Response:** We appreciate and accept your suggestion. We have edited the ‘operational definitions’ paragraph in lines 126 to 133 as per your suggestion for clarity.
